# Use of a Bio-Inductive Collagen Implant in Distal Triceps Tendon Repair: A Case Report

**DOI:** 10.7759/cureus.112320

**Published:** 2026-07-09

**Authors:** Nicholas Cummins, Christopher J Fleisher, Edward L Major, Chaitu Malempati

**Affiliations:** 1 College of Medicine, University of Kentucky, Lexington, USA; 2 Orthopedic Surgery and Sports Medicine, University of Kentucky, Bowling Green, USA

**Keywords:** bio-inductive collagen implant, distal tear, repair, tendon augmentation, triceps tendon

## Abstract

Distal triceps tendon injuries are uncommon but clinically significant, typically occurring after heavy eccentric loading and resulting in loss of active elbow extension. Complete ruptures are traditionally treated with surgical repair using transosseous tunnels or suture anchors; however, high retear rates have been reported following traditional repair techniques. Bio-inductive collagen implants have demonstrated efficacy in augmenting rotator cuff repair by promoting neovascularization and tissue regeneration; however, their use in distal triceps tendon injuries has not been previously reported. A 59-year-old male agricultural worker with a history of tobacco use presented with an acute complete distal triceps tendon rupture sustained while climbing into farm equipment. Physical examination revealed an inability to actively extend the elbow against resistance and a palpable defect over the distal triceps tendon. Magnetic resonance imaging confirmed a full-thickness tear with 1.5 cm of proximal tendon retraction from the olecranon insertion. The patient underwent open surgical repair using two suture anchors with a whipstitch technique augmented with a resorbable bio-inductive collagen implant secured over the repair site. At two weeks postoperatively, the surgical incision was well-healed without complications. By six weeks, the patient achieved active elbow motion from 10° to 90° of flexion. After three months, a full range of motion and near-normal triceps strength were restored. At one-year follow-up, magnetic resonance imaging demonstrated complete implant resorption, intact tendon continuity, and no evidence of retear. No adverse immunological reactions or postoperative complications were observed. This case demonstrates the technical feasibility and safety of bio-inductive collagen implant augmentation in distal triceps tendon repair, with excellent functional recovery at one-year follow-up. These findings provide a proof-of-concept for the use of bio-inductive augmentation in distal triceps repair and support further investigation in larger comparative studies.

## Introduction

Distal triceps tendon ruptures are uncommon injuries, representing less than 1% of all tendon injuries [1]. Associated risk factors include renal disease and anabolic steroid use [2]. These ruptures may be partial or complete, with tears most commonly occurring at the osteotendinous junction and frequently accompanied by bony avulsion [2]. While low-demand patients with partial tears may be managed conservatively with immobilization and physical therapy, full-thickness tears and high-demand patients typically require surgical intervention [3]. Traditional repair techniques utilize trans-osseous tunnels or suture anchor fixation to reattach the tendon to its olecranon insertion.

Bio-inductive collagen implants have emerged as a promising augmentation strategy for tendon repair. These resorbable scaffolds promote neovascularization and tissue regeneration, demonstrating increased tendon thickness and improved healing in rotator cuff repair [4-6]. This mechanism involves the recruitment of host cells and growth factors, creating a favorable environment for tendon regeneration. The poor healing in the area has been attributed to a lack of vascularity, which is improved by these implants. However, despite their success in rotator cuff pathology, there have been few cases of augmentation with bio-inductive implants in triceps tendon repair reported, and those that have been reported do not show a one-year MRI follow-up. Due to the implants' success in rotator cuff repair, there was reason to believe they could improve outcomes in tricep tendon repair.

We present what is, to our knowledge, the third reported case of distal triceps tendon repair augmented with a bio-inductive collagen implant (REGENETEN Bio-inductive Implant System; Smith & Nephew, Andover, MA, USA) with one-year follow-up outcomes.

## Case presentation

Patient presentation and clinical evaluation

A 59-year-old right-hand-dominant male agricultural worker with a history of tobacco use (20 pack-years) and previous methicillin-resistant *Staphylococcus aureus* infections presented with acute right elbow pain and weakness. One week prior, he sustained an injury while climbing into a combine harvester and grasping a railing for support when he felt an acute pop in his posterior elbow region. He reported an immediate onset of pain and a progressive inability to extend his elbow or lift objects.

Physical examination revealed a palpable defect approximately 2 cm proximal to the olecranon, with overlying edema and mild erythema. Active elbow extension was significantly limited, with an extension lag of approximately 30°. The patient was unable to extend his elbow against gravity or resistance. Passive range of motion was full (0-140 degrees). Neurovascular examination revealed intact radial and ulnar pulses with normal sensation in all nerve distributions. No elbow instability was appreciated.

Imaging studies

**Figure 1:**
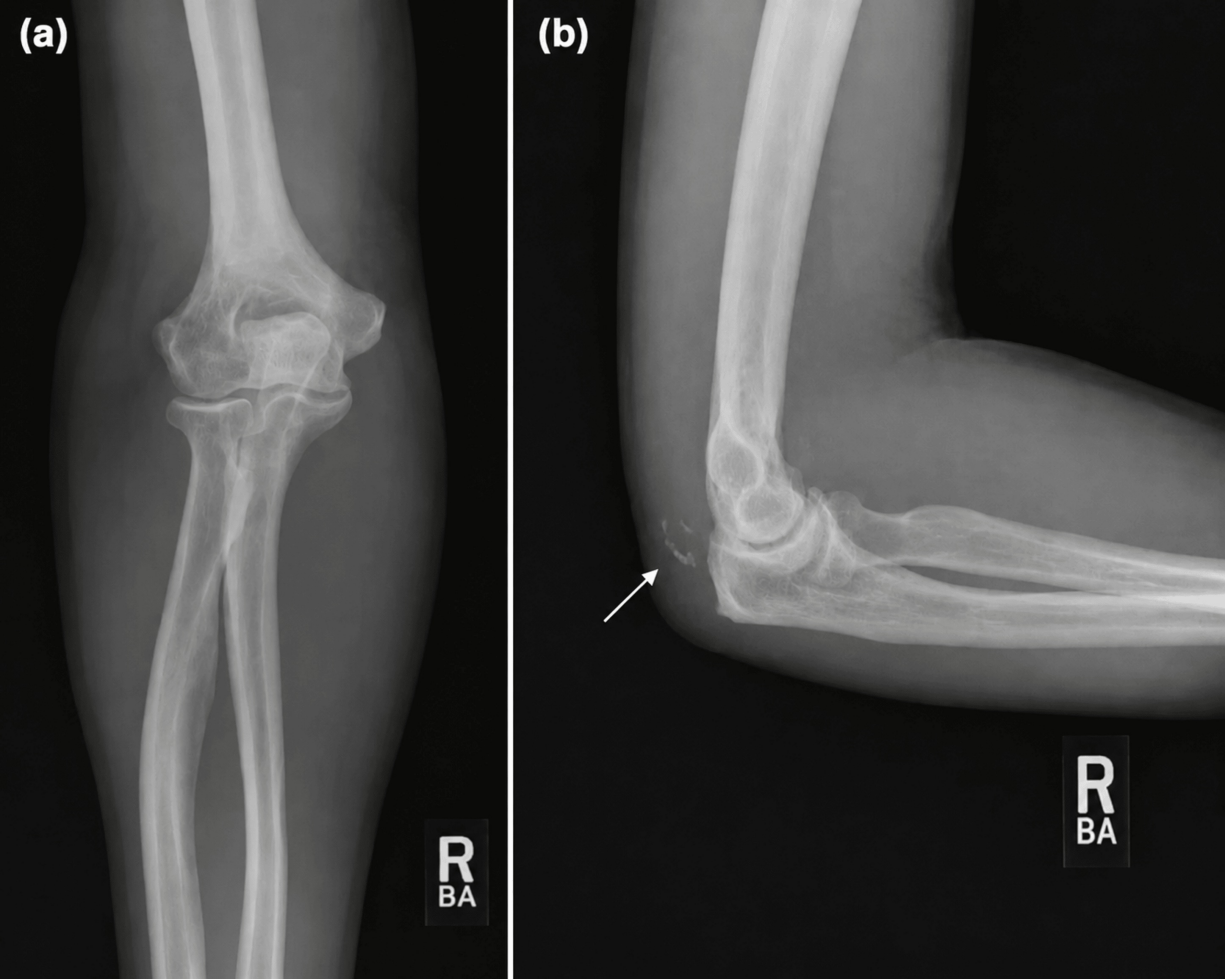
a) Anterior-posterior radiographic view of the right elbow; b) Acute flexion anterior-posterior radiograph with notable avulsion of the olecranon process

**Figure 2:**
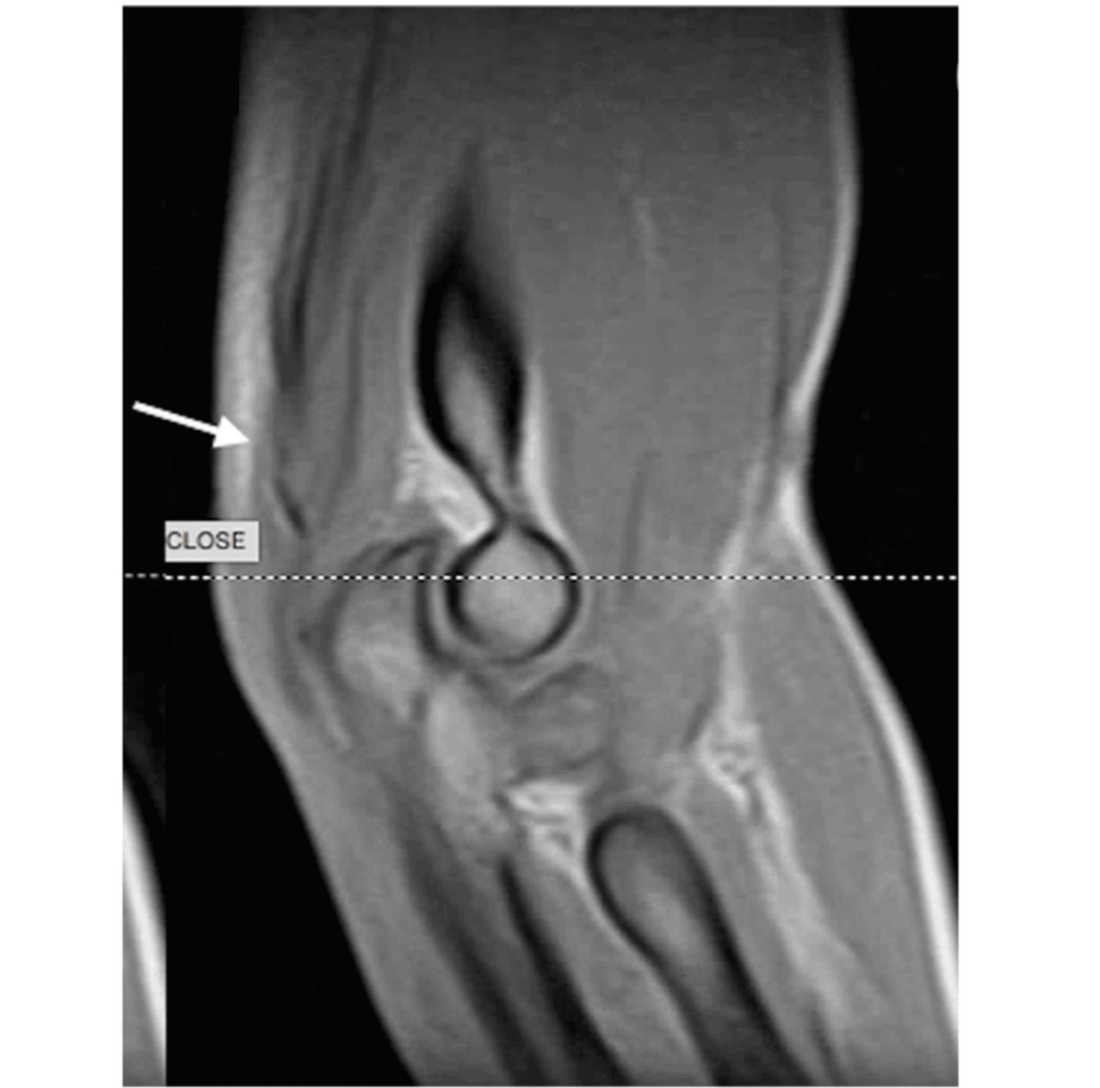
MRI showing a full-thickness triceps tendon tear with triceps muscle contracture

Two-view radiographs of the right elbow showed no acute fracture, avulsion fragment, or other bony abnormalities, consistent with a soft-tissue tendon rupture (Figure 1). Magnetic resonance imaging confirmed a complete full-thickness distal triceps tendon rupture with 1.5 cm proximal retraction from the olecranon insertion and mild edema within the distal triceps muscle belly (Figure 2). No significant muscle atrophy or fatty infiltration was noted. After discussing the treatment options, including the risks, benefits, and alternatives, the patient elected to proceed with surgical repair.

Surgical technique

On the day of surgery, the procedure was again discussed, and both the surgeon and patient agreed to proceed. The right upper extremity was marked, and general anesthesia was administered. The patient was placed in a left lateral decubitus position, draped, and a sterile size 18 tourniquet was applied to the axilla. A posterolateral approach was used, and an incision was made with a 15-blade curving laterally around the olecranon to avoid the medial side and the ulnar nerve.

A seroma consistent with the distal triceps tendon avulsion of the bone was observed. It was discovered that the tendon was completely avulsed from the bone. After debriding and irrigating adequately, two suture anchors (Q-FIX; Smith & Nephew, Andover, MA, USA) were placed in the attachment site at the olecranon process. Two sutures from each anchor were whipstitched up the tendon, and four knots were tied with tension, allowing for good repair and reduction of the triceps down to the olecranon process. Given the patient's smoking history and concern for healing potential, the resorbable collagen implant (REGENETEN; Smith & Nephew, Andover, MA, USA) was then placed over the distal tendon and sewn in using 0-vicryl sutures. Good repair and adequate range of motion were noted prior to closing of the incision. Xeroform, sterile 4x4 gauze, ABD padding, and rayon cast padding were all placed under a mold plaster splint with the arm in 60 degrees of flexion.

Postoperative management and outcomes

The patient was immobilized for two weeks and then transitioned to a hinged elbow brace locked at 30-60 degrees of flexion with supervised physical therapy. At the two-week follow-up, the surgical incision was well healed without erythema, drainage, or signs of infection. At six weeks postoperatively, brace motion was advanced to 0-90 degrees. Examination revealed active extension from 10° to 90° of flexion and no instability. The incision was well-healed without tenderness.

By three months postoperatively, the patient had regained full active range of motion (0-140 degrees) with Manual Muscle Testing grade 4+/5 triceps strength compared to 5/5 on the contralateral side. He reported minimal pain (visual analog scale 1/10) and had returned to modified agricultural duties, avoiding heavy lifting.

At the one-year follow-up, the patient demonstrated symmetric bilateral elbow range of motion and 5/5 triceps strength. The patient returned to full, unrestricted work activities without limitations. Follow-up MRI demonstrated complete healing of the tendon-bone interface, with no gap or fluid signal. The bio-inductive implant had completely resorbed with no visible remnants or distinguishable tissue boundaries. No complications, adverse reactions, or evidence of re-tear were observed during the follow-up period. Written informed consent was obtained from the patient for publication of this case and accompanying images.

At the one-year follow-up, the patient presented with a partial re-tear of the tendon after hearing a pop in his elbow while pushing down on a truck. The patient demonstrated pain behind the elbow with certain movements of the wrist or hand and difficulty with the range of motion. The injury improved after physical therapy and brace immobilization of the elbow, and the patient was able to return to work full duty.

## Discussion

This case represents what is, to our knowledge, the third reported use of a bio-inductive collagen implant for augmentation of distal triceps tendon repair and the first reporting of one-year follow-up information after such a technique. At the one-year follow-up, the patient experienced a partial retear of the tendon that was able to be managed conservatively with brace immobilization and physical therapy.

The rationale for bio-inductive augmentation in this case stems from the established challenges of triceps tendon healing. Distal triceps ruptures occur at the hypovascular osteotendinous junction, predisposing to healing complications and possible retear with traditional repair techniques. A recent systematic review showed a low retear rate of 5% using traditional trans-osseous repair methods, but smaller case series have shown higher retear rates of up to 21.4% [7,8]. Our patient's tobacco use further compromised the healing potential, prompting consideration of augmentation strategies [9]. Bio-inductive collagen implants have demonstrated efficacy in rotator cuff repair by promoting neovascularization and organized tissue regeneration [4]. Thon et al. reported increased tendon thickness and improved structural integrity on serial imaging in 23 patients with high-grade rotator cuff tears at the 24-month follow-up [5]. Similar augmentation has been described for hip abductor [10], hip capsule [11], and patellar tendon [12] repairs, suggesting broad applicability across anatomical sites with compromised healing environments.

In rotator cuff applications, this process yields increased tendon thickness and enhanced biomechanical properties [5]. The complete implant resorption observed on one-year MRI in our case, without adverse immunologic reaction, aligns with established safety profiles [13].

However, important limitations must be acknowledged. As this is a single case report, causation cannot be established; the favorable outcome may reflect natural healing with standard repair rather than implant-specific benefits. No objective strength measurements (dynamometry) or validated outcome scores (Mayo Elbow Performance Score [14], Quick Disabilities of the Arm, Shoulder and Hand [15]) were obtained, which limited the quantitative assessment. The one-year follow-up demonstrated a partial retear of the tendon, and while this retear was able to be managed conservatively with immobilization and physical therapy, reinjury is a significant concern for any tendon repair procedure. Additionally, the patient's moderately favorable outcome may reflect optimal case selection rather than generalizable results, and the lack of comparative data prevents the determination of superiority over standard repair techniques.

This case demonstrates the technical feasibility and safety of bio-inductive augmentation in distal triceps repair. Prospective comparative studies with validated outcome measures, objective strength testing, and longer follow-up are warranted to determine whether this technique reduces retear rates and improves functional outcomes compared with standard repair methods.

## Conclusions

This case demonstrates the technical feasibility and safety of bio-inductive collagen implant augmentation for distal triceps tendon repair, with moderately successful one-year outcomes and complete implant resorption. Although a single case cannot establish efficacy, this report provides proof-of-concept for augmentation at an anatomical site with high retear rates. This case suggests further studies on bio-inductive augmentation in triceps tendon repair could be useful.
